# Follicular Dendritic Cells Retain Infectious HIV in Cycling Endosomes

**DOI:** 10.1371/journal.ppat.1005285

**Published:** 2015-12-01

**Authors:** Balthasar A. Heesters, Madelene Lindqvist, Parsia A. Vagefi, Eileen P. Scully, Frank A. Schildberg, Marcus Altfeld, Bruce D. Walker, Daniel E. Kaufmann, Michael C. Carroll

**Affiliations:** 1 Program in Cellular and Molecular Medicine, Boston Children’s Hospital, Harvard Medical School, Boston, Massachusetts, United States of America; 2 Department of Microbiology and Immunobiology, Harvard Medical School, Boston, Massachusetts, United States of America; 3 Department of Medical Microbiology, University Medical Center Utrecht, Utrecht, the Netherlands; 4 Ragon Institute of Massachusetts General Hospital, Massachusetts Institute of Technology and Harvard University, Cambridge, Massachusetts, United States of America; 5 Center and Center for HIV/AIDS Vaccine Immunology and Immunogen Discovery, The Scripps Research Institute, La Jolla, California, United States of America; 6 Department of Surgery, Massachusetts General Hospital, Boston, Massachusetts, United States of America; 7 Department of Medicine, Brigham and Women's Hospital and Harvard Medical School, Boston, Massachusetts, United States of America; 8 Department of Viral Immunology, Leibniz Institute for Experimental Virology, Hamburg, Germany; 9 Centre de Recherché du CHUM; Department of Medicine, Université de Montréal, Montreal, Quebec, Canada; 10 Department of Pediatrics, Harvard Medical School, Boston, Massachusetts, United States of America; Fred Hutchinson Cancer Research Center, UNITED STATES

## Abstract

Despite the success of antiretroviral therapy (ART), it does not cure Human Immunodeficiency Virus (HIV) and discontinuation results in viral rebound. Follicular dendritic cells (FDC) are in direct contact with CD4+ T cells and they retain intact antigen for prolonged periods. We found that human FDC isolated from patients on ART retain infectious HIV within a non-degradative cycling compartment and transmit infectious virus to uninfected CD4 T cells in vitro. Importantly, treatment of the HIV+ FDC with a soluble complement receptor 2 purges the FDC of HIV virions and prevents viral transmission in vitro. Our results provide an explanation for how FDC can retain infectious HIV for extended periods and suggest a therapeutic strategy to achieve cure in HIV-infected humans.

## Author Summary

Human immunodeficiency virus (HIV) can lead to acquired immunodeficiency syndrome, or AIDS. Before the introduction of anti retroviral therapy (ART) in the mid-1990s, people with HIV could progress to AIDS in just a few years. Today patients with HIV have a close to normal life expectancy. Worldwide, there are about 2 million new cases of HIV per year. Currently about 35 million people are living with HIV of which around 13 million receive ART. Still an estimated 1.5 million people die from the consequences of HIV each year. Despite the success of ART, it does not cure HIV and discontinuation results in viral rebound. Follicular dendritic cells (FDC), located central to the B cell follicle, are also in direct contact with T cells. FDCs retain intact antigen for prolonged periods. We found that human FDCs isolated from patients on ART retain infectious HIV and can transmit virus to uninfected T cells in vitro. Treatment of the HIV+ FDC with a soluble complement receptor 2 purges the FDC of HIV virions and prevents viral transmission to T cells in vitro. Our results can explain how FDCs retain infectious HIV and suggest a therapeutic strategy to come closer to a cure.

## Introduction

Anti-retroviral therapy (ART) is capable of suppressing plasma viral load to undetectable levels and in many cases results in restoration of circulating CD4 T cell counts to near normal values. Despite the success of ART, when treatment is halted the virus rebounds suggesting the presence of a long lived reservoir [1]. Despite low viremia of circulating blood, the CD4 T cells in the LNs appear to undergo continuing infection suggesting a local source of virus. For example, characterization of lymph node (LN) biopsies of HIV infected patients undergoing ART by *in situ* hybridization (ISH) and immunohistochemistry (IHC) identify infected CD4 T cells [2–4]; and recent studies identify T follicular helper (TFH) cells as a major target of HIV [5,6]. Further support for LNs as a major site for continued infection of CD4 T cells was reported recently in non-human primates. Simian Immunodeficiency Virus (SIV) rapidly seeds the reservoir in LNs even before detectable systemic viremia [7]. Together, these observations suggest that cells in lymphoid organs, which are not reflected in systemic measures of viral load, are among the first to become infected and constitute the initial reservoir.

While latency in CD4 T cells represents one possible source of persisting virus [8–10], an additional reservoir are follicular dendritic cells (FDC). FDCs, which are located central to the B cell follicle, are a source of the chemoattractant (CXCL13) for B cells and TFH, and are required for maintenance of follicle structure [11,12]. They are stromal derived and long recognized for their ability to retain antigen as an immune complex (IC) for periods of at least one year in mice [13,14]. They can trap complexes of complement -opsonized virus through the CD21 receptor much like IC [15–17]. The presence of viral RNA co-localizing with FDC suggests the stromal cells may serve as a continuing source of infectious virus; however, their role as a potential reservoir or depot has not been fully explored [18,19]. Thus, it is possible that infectious virus is retained in a non-replicating form by FDC making it accessible to CXCR5^+^ CD4^+^ TFH as they traffic into the B cell compartment.

HIV is capable of independently fixing complement through complement factor I and, paradoxically, this enhances HIV infectivity in vitro [20–22]. Moreover, the recent finding that HIV coat proteins contain mannose groups suggests the lectin pathway of complement could participate via mannan binding protein in opsonization of viral particles [23]. Alternatively, HIV specific antibodies can, through the classical complement pathway, activate and deposit complement on the viral surface [24,25]. We propose that complement-opsonized HIV might exploit the recently identified mechanism of FDC uptake and cycling of IC in a non-degraded state for extensive periods [26]. Notably, the earlier studies found that IC opsonized with complement C3d are internalized via CD21 receptor into a cycling, non-degradative endosomal compartment that co-localizes with the transferrin receptor. Thus, the findings suggest a mechanism by which infectious virus can be retained within a neutral pH compartment inside the cell but periodically cycled to the cell surface for infection of CD4 T cells [27]. This mechanism could provide a safe-haven for the non-dividing virus, allowing it to escape both ART and detection by the immune system while remaining infectious. We sought to test this hypothesis by specifically determining the ability of FDCs isolated from LNs of HIV- and HIV+ subjects on prolonged ART to carry infectious virions. We show that human FDCs cycle antigen and retain infectious HIV but, unlike CD4 T cells, do not harbor provirus in their genome. Strikingly, the virus can be displaced by treatment with a soluble form of human CD21 receptor fused to murine IgG1 (sCD21-Ig). Thus, treatment of HIV+ FDC with sCD21-Ig prevents infection of healthy CD4 T cells *in vitro*.

## Results

### Human FDC internalize and cycle immune complexes

**Fig 1:**
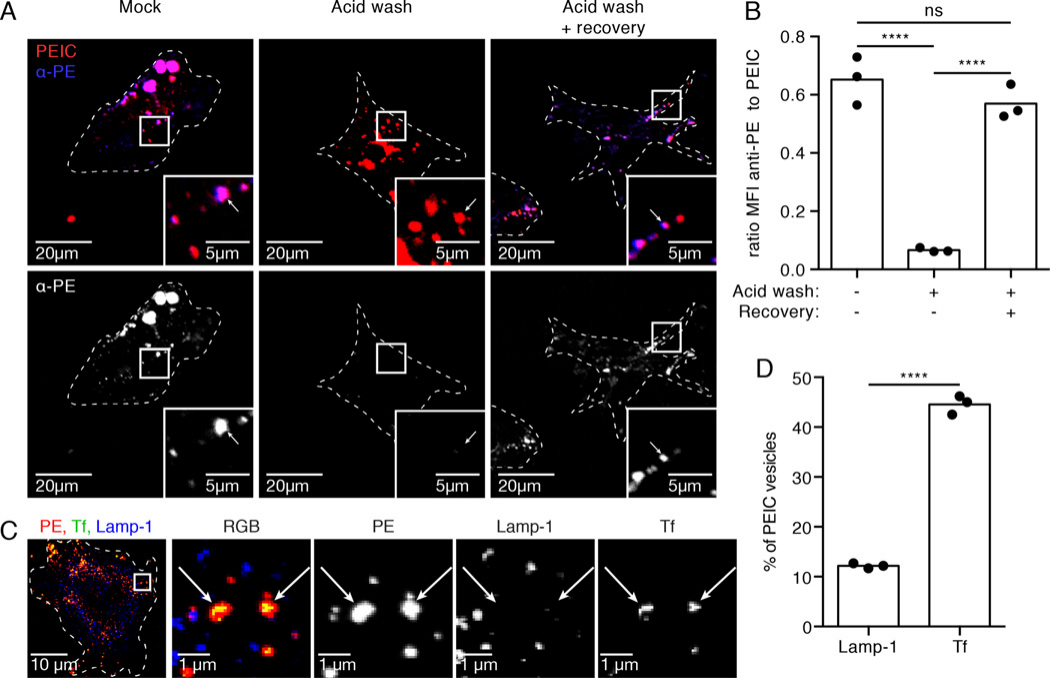
Human FDCs recycle immune complexes in a transferrin (Tf) positive non-degradative endosomal compartment. (A) Human FDCs from lymph node biopsies of HIV-negative subjects were loaded with complement C3-opsonized phycoerythrin immune complexes (PEICs), then either fixed (left), acid washed and fixed (middle), or acid washed, incubated in media for 30 min (recovery), and then fixed (right). Fixed FDCs were surface stained with an antibody against PE. PEICs detected on the surface of the FDC after acid wash and recovery must have come from the inside of the cell since no PEIC was detected on the surface after the acid wash. (B) Quantification of the images shows efficient stripping of PEICs by acid wash treatment and a robust recovery of PEICs on the surface after 30 min. ****P<0.0001 (Two-way ANOVA, multiple comparisons) n = 3 (3 subjects, 4 replicates each). (C) On human FDCs PEIC reside mainly in Tf positive compartments, as is the case in mice. The recycling Tf compartment was visualized by incubation of live cells with fluorescent Tf for 8 minutes. (D) The percentage of Tf or Lamp-1 positive vesicles within the PEIC positive vesicles was quantified. This shows ~45% of PEICs in Tf positive compartments and only ~12% in Lamp-1 positive compartments. ****P<0.0001 (Two-way ANOVA) n = 3 (3 subjects, 6 replicates each).

To determine if human FDCs take-up and cycle antigen similar to murine FDCs, complement C3 opsonized phycoerythrin immune complexes (PEIC) were used as a model antigen [26]. The fluorescence characteristics and the availability of specific antibodies make PEICs easy to track inside and outside the cell. PE was incubated with human serum and anti-PE antibody, the anti-PE antibodies bind PE, leading to activation of complement components in the serum and subsequent opsonization with C3. A single cell suspension of cells was prepared from LNs of healthy donors and FDC were positively selected to approximately 85% purity using a combination of anti-CD45 to negatively select the LN stromal population followed by positive selection with anti-CD35 magnetic beads. Purified FDCs were cultured on collagen-coated coverslips for four days, until the FDCs regained their dendritic morphology. Naïve, non-cognate, allogeneic B cells were loaded with PEICs, subsequently these B cells with surface bound PEIC were added to the FDC cultures in order to mimic *in vivo* conditions and select for successfully opsonized PEIC. FDCs acquire the PEIC from the donor B cells in a CD21-dependent manner [26]. Following co-culture with B cells, the FDC cultures were fixed with or without an acid wash treatment that strips the surface of PEIC. Allowing a recovery period after acid wash and before fixation allows restoration of surface levels from an intracellular source (Fig 1A). To quantitate PEIC on the cell surface versus total cellular levels, the ratio of the mean fluorescence intensity (MFI) of anti-PE to total PEIC was determined for each FDC (Fig 1B). The acid wash treatment efficiently stripped the FDC surface of PEIC (mean±SD ratio MFI anti-PE:PEIC, 0.06±0.01) and after recovery PEIC was detected on the surface of the FDC (0.57±0.04) in a similar ratio as without acid wash (0.67±0.08). These data indicate that the PEIC was protected inside the cell during acid wash but cycled to the surface during recovery phase. These data show that human FDCs, similar to their murine counterparts, cycle immune complexes.

To examine whether model IC are taken up and transported into the cycling or the lysosomal compartment, transferrin (Tf) and Lamp-1, respectively were used as endosomal markers. Lamp-1 primarily localizes with the degradative compartment, whereas Tf localizes mainly with the neutral pH recycling endosomal compartment [27]. The majority (mean±SD, 44.6%±1.9%) of PEICs resides in vesicles co-localized with Tf staining whereas only a small percentage (12.2%±0.5%) was associated with Lamp-1 vesicles (p<0.0001). This indicates that the majority of PEIC cycle through a non-degradative endosomal compartment, while only a fraction of the PEIC is being degraded (Fig 1C and 1D). This observation is in line with earlier observations in mice where IC are degraded at early time points, until a steady state is reached [14,26]. In short, upon binding the FDC there are two potential fates for the IC: degradation in a lysosomal compartment or retention in a periodically cycling endosome that bears a neutral pH [27]. Over a period of time, a stable state is reached in which the antigen retention remains at a constant level. Thus, human FDC appear to continuously cycle the majority of the antigen to their surface in a non-degradative endosomal compartment, similar to the pattern observed in murine FDC loaded with IC *in vitro* [26].

### Human FDC internalize HIV *in vivo*

A hallmark of FDCs is their ability to retain antigen for extensive periods in the form of an IC [28–30]. Earlier studies suggest that HIV is retained similar to IC by FDC in LNs [16,31]. Histology of LNs isolated from HIV subjects and stained for HIV antigen or RNA indicates a reticular pattern that co-localizes primarily with FDC dendrites [19,31,32]. Although mice are not infected with HIV, many of the other components of the immune system, like complement, are in place and isolation of FDC from LNs of mice injected with opsonized HIV indicate retention of infectious virus for periods up to nine months based on transmission of virus [16,33]. However, actual visualization of virus co-localizing on FDC was not reported; nor was it clear how the virus was retained [16,34]. To test if HIV is transported and retained by FDCs in a murine model, mice were injected with a recombinant form of HIV (HIV-GFP) bearing GFP. Draining LNs were harvested 48 hours later for cryosections and imaging. Analysis of LN sections by scanning confocal microscopy identifies co-localization HIV-GFP virus with FDC dendrites similar to that observed with PEIC (S1 Fig). Thus, in the absence of a productive infection of murine CD4 T cells, HIV-GFP becomes opsonized *in vivo* and is transported to the FDC within the follicles of the draining LNs.

To assess whether human FDC retain HIV and localize it to a similar compartment as observed with PEIC, inguinal LN tissue was harvested from seven HIV+ subjects classified as chronic progressors (CP) and undergoing therapy with ART and three healthy controls (Table 1). FDCs were isolated using a two-step procedure as noted above. Although visualization of the cultured cells after purification showed an absence of lymphocytes, it was important to limit carry-over of contaminating T cells in the FDC cultures especially since there is evidence of a subpopulation of human CD4 T cells that express CD35. Analysis of CD4 T cells isolated from HIV+ subjects by flow cytometry identified a minor population that expressed low levels of CD35 relative to B cells. However, sorting of a mixture of CD4 T and B cells isolated from an HIV+ subject by immuno-purification with anti-CD35 confirmed negligible presence of T cells (S5 Fig).

**Table 1:** Patient data. Phenotype negative represents healthy subjects. CP phenotype is chronic progressor. ARV (anti-retroviral). CD4 and CD8 count are per ml of blood. Serum viral load was determined one week before surgery using the hospital standard method, which has a detection limit of 20 virions/ml.

**Patient**	**Phenotype**	**CD4 Count (%)**	**CD8 Count (%)**	**Viral Load**	**# Viral Blips**	**ARV (yr)**
1	Negative	---	---	<48	n/a	n/a
2	Negative	---	---	<20	n/a	n/a
3	Negative	---	---	<48	n/a	n/a
4	CP	635 (32)	926 (47)	61	1	1
5	CP	1371 (33)	1814 (44)	<20	5	10
6	CP	712 (35)	717 (37)	<20	7	24
7	CP	458 (23)	838 (41)	<20	0	8
8	CP	667 (24)	--- (39)	<20	5	7
9	CP	1083 (48)	--- (26)	<20	1	6
10	CP	419 (27)	687 (44)	<20	1	13

CP = Chronic Progressor, n/a = not applicable, ARV = Anti RetroViral

**Fig 2:**
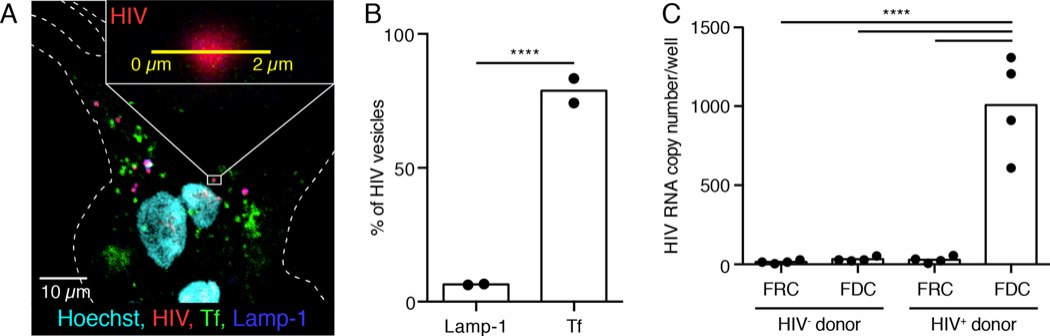
Human FDCs isolated from HIV positive subjects on ART treatment retain HIV in a Tf positive compartment. (A) Cultured human FDCs from HIV+ subjects on ART were stained for Tf (green), HIV (p24, red), Lamp-1 (blue) and Hoechst (cyan). (inset) A single vesicle was enlarged and only the red (HIV) channel was shown. The yellow line was used for line profile analysis, which measures the fluorescent intensity over that line in all channels. Line profiles were also made in the z direction to ensure co-localization of HIV+ vesicle in the X, Y and Z direction with Tf or Lamp-1. (B) Quantification of line profile measurements of HIV positive vesicles. The vast majority of HIV containing vesicles were positive for Tf (~80%), while only ~5% was positive for Lamp-1 ****P<0.0001 (Two-way ANOVA) n = 2 (2 subjects, 4 replicates each). (C) FDCs and FRCs from HIV positive individuals on ART treatment and HIV negative subjects were cultured and viral RNA levels were quantified. RNA levels are represented per well. FRC and FDC were isolated from the same subjects to control for contaminating cells. Each data point presents an individual subject. ****P<0.0001 (Two-way ANOVA, multiple comparisons) n = 4 (4 subjects, 3 replicates).

Enriched FDC were cultured for 5 days to regain their dendritic morphology before imaging. HIV was detected in FDC stromal cells by confocal microscopy analysis of permeabilized cells after staining with KC-57 antibody specific for the HIV core protein p24 (Fig 2A and 2B, S2 Fig). A mean of 23% of FDCs were positive for HIV antigen and of these, a range of 1.4 to 2.9 vesicles per FDC were positive for p24 antigen (Table 2). HIV-containing vesicles co-localized in 3 dimensions with transferrin, but not with Lamp-1 (Fig 2A and 2B; Table 2). Thus, despite the prolonged ART (1 to 24 years, median = 8 years) a fraction of the FDC remained positive for HIV antigen in all subjects examined. HIV is primarily detected within the cycling endosomal compartment (mean±SD, 78.8%±6.5%), and not in the lysosomes (6.5±0.3%). A similar pattern was observed in FDC isolated from LNs of mice passively immunized with PEIC where a negligible level of the IC+ vesicles co-localized with the Lamp-1 compartment [26]. Finding that HIV co-localized with transferrin was important as this compartment is relatively neutral, i.e. pH 6.0–6.4; and therefore unlikely to inactivate the virus which is inactive at pH 5.4 and lower [27,35].

**Table 2:** Table summarizes number of FDCs analyzed by confocal per patient sample. The number of FDCs per well was calculated by counting FDCs in multiple fields of view (FOV) within a known area. Based on this area and the total surface area of the well, an estimate of total FDCs per well was calculated. Staining FDC with anti-p24 and counting HIV positive vesicles determined positive cells. The table compares the estimated HIV positive vesicles per well based on imaging with the average RNA copy number found per well in the corresponding subjects.

**Patient ID**	**FDCs/well(estimated)**	**# of FDCsanalyzed**	**% HIV** ^**+**^ **FDC**	**#HIV** ^**+**^ **vesicles**	**HIV** ^**+**^ **vesicles/HIV** ^**+**^ **FDC**	**HIV** ^**+**^ **vesicles/well**	**HIV RNACopy#/well**
1	10k-100k	112	0	0	0	0	2.4
2	4k-40k	48	0	0	0	0	5
3	7-70k	79	0	0	0	0	2
4	8k-80k	86	19.8	24	1.4	2k-20k	1620
5	5k-50k	56	12.5	12	1.7	1k-10k	609
6	5k-50k	68	16.2	17	1.5	1k-10k	1204
7	2k-20k	-	-	-	-	-	910
8	4k-40k	-	-	-	-	-	709
9	3k-30k	36	22.2	23	2.9	2k-20k	1422
10	15k-150k	187	46.0	206	2.4	15k-150k	1596

In order to determine if human FDC retain viral RNA in addition to viral protein, cellular RNA was prepared from enriched FDC harvested from HIV+ and HIV- subject LNs and analyzed by droplet digital RT-PCR (ddPCR). The viral copy number was determined per well. As expected, no viral RNA was observed in FDCs from HIV- subjects. By contrast, FDC isolated from HIV+ subjects retained significant levels of viral RNA, i.e. mean±SD, 1007±314.3 viral copies per well of FDC (Fig 2C and Table 2). These *ex vivo* cultures are enriched for FDCs but potentially include fibroblast reticular cells (FRC) and contaminating HIV infected CD4+ T cells. Although no contaminating CD4 T cells were detected in the cultures as discussed above, the FDC extract was assayed for the presence of CD4 RNA. The results from ddPCR confirmed the absence of contaminating CD4 mRNA in the FDC *ex vivo* cultures (S6 Fig). To test if FRC, which are the most likely contaminant in the FDC cultures, are a possible reservoir for HIV, FRCs were purified and cultured from the same LNs as the FDC and RNA was isolated. Analysis of RNA extracted from the cultures of FRC showed negligible viral RNA suggesting that contaminating FRC are unlikely to explain the presence of viral RNA in the FDC cultures (Fig 2C). Moreover, co-culture of FRC with healthy CD4 T cells showed no transmission of virus.

### Infectious HIV is retained by FDC via complement receptor CD21

**Fig 3:**
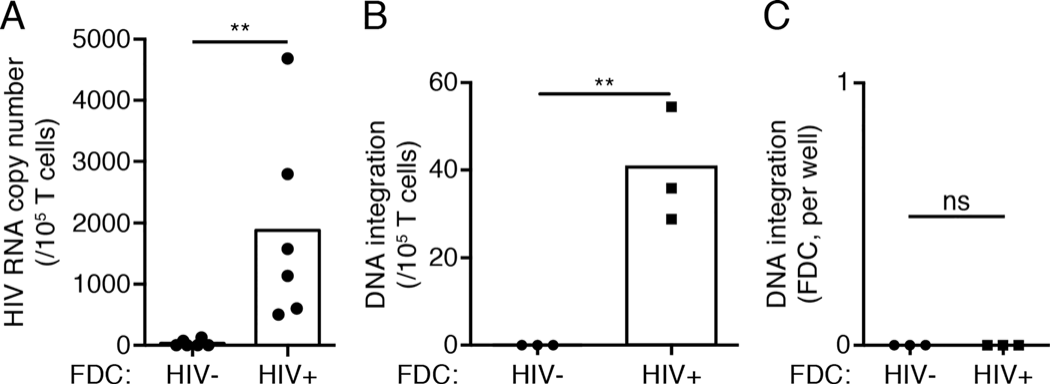
FDCs are a source of HIV infection for T cells. FDC cultures (10^3^ to 10^5^ cells) of HIV positive subjects on ART were co-cultured with activated CD4 T cells (10^5^ cells) from PBMC of healthy subjects for 5 days. Non-adherent CD4 T cells were removed from the co-cultures by washing and analyzed for viral RNA and DNA content. (A) RNA from CD4 T cells was collected for analysis. T cells co-cultured with FDCs from HIV+ subjects contained viral RNA, in contrast to the T cells co-cultured with FDCs from healthy subjects. This indicates transfer of HIV from FDC to T cell. Each data point presents an individual subject. **P<0.01 (Two-way ANOVA) n = 6 (6 subjects, 2 replicates each). (B) DNA from CD4 T cells was collected for analysis. Integrated viral DNA was detected in T cells co-cultured with FDCs from a HIV+ subject, in contrast to the T cells co-cultured with FDCs from a healthy subject. This indicates productive infection of T cell. DNA integrations normalized to 10^5^ T cells. **P<0.01 (Student’s *t* test) n = 1 (1 subject, 3 replicates). (C) DNA from FDCs was collected for analysis. FDCs from a HIV+ subject had no viral DNA integrated in their genome, just as FDCs from a healthy subject. DNA integration represented as total per well. ns = not significant (Student’s *t* test) n = 1 (1 subject, 3 replicates).

To determine if HIV retained by FDCs is infectious, FDCs isolated from ART treated HIV+ or healthy HIV- subjects were co-cultured with purified activated CD4 T cells prepared from PBMC of healthy volunteers. After 5 days of co-culture, CD4+ T cells were separated from the adherent FDC and RNA was extracted from both populations of cells (S4 Fig). Analysis of viral RNA from T cells co-cultured with HIV+ FDC identified presence of HIV RNA, i.e. mean±SD, 1883±1605 viral copies per 1 x 10^5^ T cells (Fig 3A). Moreover, DNA from CD4 T cells co-cultured with HIV+ FDC from one volunteer was analyzed for viral DNA integration (mean±SD, 36.9±12.2 integration events per 1 x 10^5^ T cells). This analysis confirmed viral integration in the genomic DNA of healthy CD4 T cells after co-culture with HIV+ FDCs (Fig 3B). Notably, the HIV+ FDCs, which retained approximately 1000 virions, did not have viral integration in their genomic DNA as expected (Fig 3C). Thus, FDC, unlike known reservoirs for HIV, do not retain HIV provirus in agreement with earlier studies [33,36]. As expected, CD4+ T cells co-cultured with FDC isolated from HIV- subjects were negative for HIV RNA and DNA. Thus, FDCs isolated from ART treated patients not only retained viral RNA but were able to transmit virus to healthy CD4+ T cells.

Complement receptor CD21 is expressed primarily on B cells and FDCs in both human and murine tissues. Earlier studies identified an important role for CD21 in HIV retention in murine lymphoid tissues and in transmission of virus to human CD4 T cells [34]. Kacani et al. reported that antibody to CD21 treatment of HIV+ tonsillar sections *in vitro* could strip a significant level of viral antigen from the follicular region [31]. By contrast antibodies to CR1 (CD35) or CR3 (CD11b/CD18) had no appreciable affect. Likewise, human B cells have been identified in the circulation of HIV+ patients with C3-opsonized HIV bound to their surface [37]. Treatment of HIV+ B cells with a similar blocking antibody releases the HIV particles, suggesting that CD21 binding to C3d is a major mechanism for viral uptake [37].

**Fig 4:**
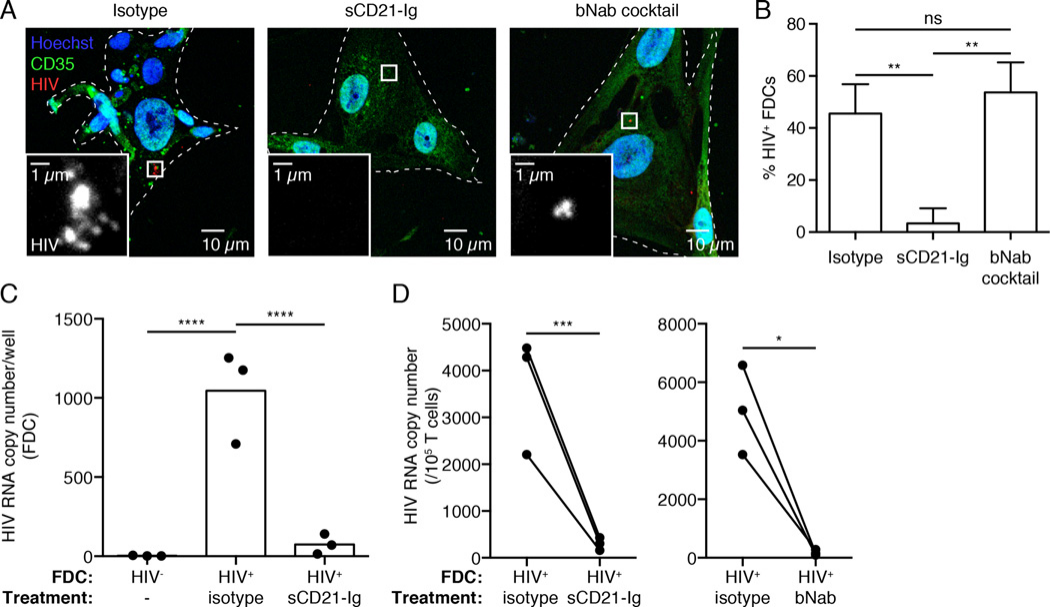
sCD21-Ig can purge HIV from the FDC and prevent infection of CD4 T cells. FDCs were isolated from LNs of HIV positive subjects on ART and cultured for 4 days. CD4 T cells from HIV negative subjects were activated in parallel. On day 4 FDC cultures were treated overnight with an isotype control antibody, soluble CD21-Ig (sCD21-Ig) or a cocktail of 3 broadly neutralizing antibodies (bNab; VRC01, PG16 and PGT121). Then cultures were washed and co-cultured for 5 additional days with 10^5^ activated CD4 T cells. On day 5 samples were split into 3 groups: FDCs for imaging (A, B), FDCs for RNA quantification (C) and T cells for RNA quantification (D). (A) FDCs were fixed, stained for p24 (HIV, red), CD35 (FDC, green) and Hoechst (blue) and imaged by confocal microscopy. Virions were detected in the isotype and bNab cocktail treated cultures, but not in the sCD21-Ig treated culture. (B) Percentage of HIV containing FDCs. 10 random field of views were collected and quantified per sample. ns: not significant, **P<0.01 (Two-way ANOVA, multiple comparisons) n = 3. (C) HIV RNA quantification of FDCs after sCD21-Ig treatment. Each data point represents an individual subject. Total HIV RNA per well is depicted. ****P<0.0001 (Two-way ANOVA, multiple comparisons) n = 3 (3 subjects, 3 replicates each). (D) HIV RNA quantification of T cells incubated with FDCs treated with sCD21-Ig (left panel) or bNab cocktail (right panel). HIV RNA is depicted per 10^5^ T cells, which is the total per well. *P<0.05, ***P<0.001 (Two-way ANOVA) n = 3 (3 subjects, up to 3 replicates).

To determine if human FDC retain HIV via CD21, FDCs positive for virus were cultured overnight with either a CD21 decoy receptor, i.e. fusion protein of the C3d binding domain of human CD21 and murine immunoglobulin, (sCD21-Ig), or an isotype control [38]. To control whether this treatment would disrupt the cycling mechanism, healthy human FDCs were loaded with PEIC and treated with sCD21-Ig. Overnight treatment with sCD21-Ig completely stripped human FDCs of PEIC (S3 Fig). When HIV+ FDC were treated with sCD21-Ig, levels of both virus antigen and RNA were reduced to background relative to the isotype control (Fig 4A–4C). FDC from HIV+ subjects treated with isotype control antibody had viral antigen present (mean±SD, 45.6%±11.3%), in contrast with FDCs treated with sCD21-Ig (3.3%±5.8%). Furthermore, the RNA copy number in sCD21-Ig treated FDCs dropped over 10 fold (mean±SD, 74.7±63.1) when compared to isotype treated FDCs (1046±294.2). Although the RNA levels in the sCD21-Ig treated group are still higher than in FDCs from HIV negative subjects (3.1±1.6), this difference is not significant. Interestingly, FDCs treated with a cocktail of broadly neutralizing antibodies (bNab) retained viral antigen (53.7%±11.6%) (Fig 4A and 4B). These results demonstrate that FDCs retain HIV in ART treated patients primarily via CD21, and that sCD21-Ig can “purge” FDCs of virus *in vitro*. Moreover, they provide further support that retention of antigen in the *ex vivo* cultures is primarily via CD21 expressing cells. We hypothesize that sCD21-Ig competes for CR2 binding of C3d on the virion, which is then released in the media. Analysis of the supernatant by ddPCR yielded no results; however, there could be numerous explanations for the inability to detect the virus such as the virus is highly labile once it is released from the FDC. Although the exact mode of action has not been shown, treatment with sCD21-Ig empties the cycling compartment of FDCs and removes HIV from the system.

### Treatment of FDC with soluble CD21-Ig blocks transmission of infectious virus

**Fig 5:**
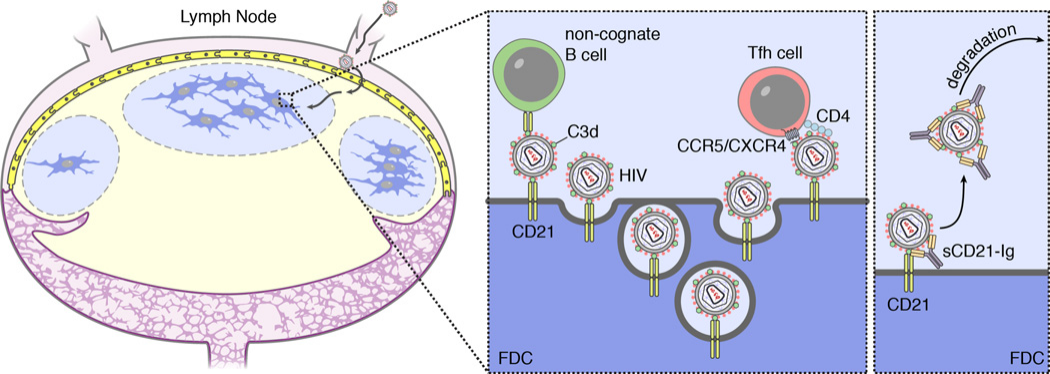
Model of FDC HIV retention, transfer to T cells and sCD21-Ig purging. Schematic model. HIV is captured and subsequently cycled by complement receptor 2 (CD21) on the follicular dendritic cell (FDC). The HIV virion resides in the protective recycling endosome of the FDC. Upon emerging from the endosome at the cell surface, HIV can infect surrounding T follicular helper (Tfh) cells that have been attracted to the FDC by a CXCL13 gradient. Infection occurs through binding of CD4 and either CXC-chemokine receptor 4 (CXCR4) or CC-chemokine receptor 5 (CCR5) as a co-receptor by gp120. The soluble CD21 receptor fusion protein (sCD21-Ig) competes with the CD21 on the FDC for binding of complement C3d on the virion. This facilitates the release of the virion and makes it available for degradation by other cells. To prevent infection of T cells at this stage the treatment should be combined with broadly neutralizing antibodies (bNab).

To test whether blockade of C3d binding by CD21 could prevent transmission of infectious virus, sCD21-Ig was added to FDC cultures that were then co-cultured with uninfected CD4 T cells as described above (Fig 3). Notably, treatment of FDC cultures with sCD21-Ig reduced viral infection of CD4 T cells significantly (mean±SD 299±134) compared to isotype control treatment (mean±SD, 3656±1261) (Fig 4D, left panel). *In vivo*, this approach could reduce infection of T follicular helper cells (TFH) by acting on two levels. Transfer of HIV from B cell to TFH or transfer of HIV from FDCs to CD4 T cells could be disrupted by sCD21-Ig, which acts to “purge” or displace C3-opsonized viral complexes from CD21+ B cells and FDC (Fig 5).

As further evidence that FDCs retain infectious HIV and as a control for viral transmission to healthy CD4 T cells, a cocktail of bNab was added to HIV+ FDC cultures prior to co-culturing with CD4 T cells. The bNabs used (VRC01, PG16 and PGT121) block CD4 binding to gp120 (VRC01) and gp120 glycans (PG16 and PGT121) [39]. As expected, the bNab cocktail, which will not block CD21 binding to C3d, failed to purge FDC of viral antigen (Fig 4B). By contrast, treatment of FDCs with bNabs reduces transmission of virus to CD4 T cells to a near background level of detection (mean±SD, 177±93) compared to treatment with isotype antibody (mean±SD, 5049±1526) (Fig 4D). This suggests that the virions are accessible to bNabs during the overnight treatment and this neutralizes the virions preventing transmission of the infectious virus in the co-culture. The FDC storage compartment might be accessible to the fluid phase or it might be exposed during recycling. The purging of the human FDC is likely not FcR mediated since the addition of neither human bNab nor the murine isotype control IgG1 eliminated the retention of virus (Fig 4B).

## Discussion

Despite the success of ART in reducing HIV viral loads and partial restoration of circulating CD4 T cells, cessation of the drugs results in acute viral rebound, suggesting the importance of a viral reservoir [40,41]. While it is generally held that latently infected CD4 T cells are a major reservoir for HIV, there is growing evidence that LNs are partly refractory to ART and include one of the earliest seeded reservoirs [7,42]. Analysis of LNs in HIV infected subjects using *in situ* RNA hybridization and immunohistochemistry specific for HIV have identified a dendritic pattern of viral deposition that co-localize with FDC in the B cell follicles [32]. These observations along with others showing that FDC extracts are positive for infectious HIV [15], raised the question whether FDC harbor infectious HIV virus and if so how is the virus retained? To address these questions, FDCs were isolated from LNs that were surgically removed from seven infected subjects undergoing long term ART and three non-infected healthy donors. All subjects had low to undetectable plasma viral loads.

It was important to first test if human FDC retain model antigen IC similar to that observed in mice. Using PEIC opsonized with complement, human FDC were loaded with the complexes in the *ex vivo* cultures. We found that the complexes were retained primarily in the cycling early endosomal compartment marked by the transferrin receptor as observed in murine FDC [26]. Thus, human FDC appear to take-up IC via CD21 receptor and cycle the complexes periodically to the cell surface where they are accessible to sampling by cognate B cells. Although, approximately 25% of the PEIC co-localized with the lysosomal compartment, this was not unexpected and consistent with our observations where murine FDC are loaded with C3 opsonized PEIC in vitro. Our current view is that FDC become saturated with excess IC when loaded *in vitro* in contrast to uptake *in vivo*. Characterization of FDC isolated from HIV+ subjects in *ex vivo* cultures identified presence of viral antigen (p24) in about 23% of the cells and of these positive vesicles, the majority co-localized with Tf ligand. Notably, negligible levels co-localized with the Lamp-1 compartment as predicted from earlier studies with murine FDC loaded with foreign antigen *in vivo* [26].

The number of viral antigen+ vesicles per FDC is in a similar range as that identified for viral RNA copies, i.e. 1.4 to 2.9 HIV+ vesicles compared to approximately 0.2–2.1 viral copies of RNA. Earlier studies of LNs of HIV+ subjects following initial treatment with ART identified a dramatic reduction in viral load in both CD4 T cells and FDC within the first a month [19,42]. In the current study, all of the HIV+ subjects showed a low level of infection (<20 copies) in their circulating CD4 T cells as expected. Moreover, analysis of viral RNA extracted from LN T cells of one HIV+ subject identified a mean of two copies of virus per 10^5^ T cells (S6C Fig). Therefore, it was not surprising that a low viral load was observed for their FDC. In agreement with the earlier findings, HIV RNA was identified, but not proviral DNA [36]. Despite not being infected, FDCs are a source of infection for CD4 T cells. Dendritic cells (DC), which are unrelated to the stromal derived FDC, are also capable of recycling HIV and form a source of infection for CD4 T cells. However, unlike FDCs, DCs are not long lived and thus cannot retain antigen for extensive periods.

Trapping of HIV on the FDC by immune complexes includes incorporation of the infectious virus into periodically cycling endosomes that retain the virus within the FDC. This not only further identifies the B cell follicle as an important reservoir of infectious virus, these data imply that the follicle can be a source of infectious virus for, potentially, several years after the individual is put on ART and has undetectable viremia in the blood.

A prediction about an HIV reservoir is that it will include multiple variants of HIV collected over an extended period. Therefore, one would expect FDC to act as an archive for multiple genotypes of virus. Earlier studies by Burton and colleagues compared the RNA sequence of *pol* and *env* genes of RNA isolated from FDC extracted from postmortem tissues with ante mortem PBMC isolated at 22, 20, 18 and 4 months prior to death [15]. Strikingly, they found near identical genotypes in FDC matching those identified at 22 months antemortem PBMC; and the virus retained infectivity. Moreover, they identified increased diversity in HIV sequence isolated from LN FDC relative to other postmortem tissues such as bone marrow, T cells and spleen [15]. Their interpretation of the results was that FDC act as an archive for variants taken-up over an extended period. Using a non-permissive mouse model, Smith et al. injected mice with isolates of HIV opsonized with antibody [33]. Periodically, draining LNs were harvested and FDC assayed for retention of infectious virus. Notably, the half-life of FDC retention of infectious virus was about 2 months. Since murine T cells are non-permissive for infection, their results provide further support for FDC retention of virus for extended periods. However, their study did not provide a mechanism for how virus was taken-up and retained by FDC in a protected manner.

In the current study, our finding that FDC take up virus via CD21 and cycle the complex within the transferrin Tf compartment provides a mechanism how FDC might serve as an archive. Tf marks a mildly acidic compartment in contrast to the lysosomal compartment and would provide a “non-labile” environment in which the virus can remain infectious for an extended period yet maintain periodic contact with TFH within the LN follicles.

There is a growing literature to support ongoing replication in lymphatic tissues of patients on ART with suppression in blood [42–44]. The number of latently infected cells does not decrease over time with ART suggesting this reservoir is replenished. Our study combined with data of Connick et al. that show cytotoxic T lymphocyte exclusion from the follicle suggests that infectious virions, when cycled to the surface of FDCs could infect TFH cells, creating a cycle of infection and replication in a privileged compartment [45].

Since the FDC cultures could potentially be contaminated with an alternative cellular source of HIV, it was important to rule this out. It was especially important to test for the presence of contaminating HIV+ CD4 T cells. Several lines of evidence make this possibility unlikely. First, LN stromal cell isolation includes a CD45 negative sort that eliminates most bone marrow derived cells, including T cells. The second step, which is positive selection with anti-CD35, further limits the possibility of T cell contamination. As an aside, we did observe a small population of CD35 ^low^ CD4 T cells among the lymphocytes isolated from an HIV infected subject. Immuno-sorting of the cells under similar conditions used for FDC determined that the CD35 positive selection step failed to enrich for the T cells probably due to the very low level of surface antigen expression (S5A and S5B Fig). Secondly, T cells isolated from the same LN as FDC showed a negligible level of viral RNA, i.e. mean of 2 copies per 10^5^ T cells based on ddPCR. Finally, no detectable CD4 RNA was observed in the FDC extracts (S6A and S6B Fig). Since FDC isolates are often contaminated with FRC, which are about 100 fold more abundant in the LN, it was important to rule them out as a possible source of HIV [29]. In cultures of FRC isolated from HIV+ subjects using a similar protocol as for FDC no detectable virus was found.

Previous studies support ongoing replication in lymphatic tissues of patients on ART with viral load suppression in blood [42–44]. The number of latently infected cells does not decrease over time with ART suggesting this reservoir is replenished. Our study combined with data of Connick et al. that show cytotoxic T lymphocyte exclusion from the follicle suggests that infectious virions, when cycled to the surface of FDCs could infect TFH cells, creating a cycle of infection and replication in a privileged compartment [45].

Earlier studies in mice identified the importance of CD21 receptor in uptake and retention of foreign antigen by FDC [29,46]. To determine if uptake of HIV virus is also dependent on CD21, *ex vivo* cultures of FDC isolated from HIV+ subjects were treated overnight with a fusion protein bearing the C3d binding site of the human CD21 receptor and a murine IgG (sCD21-Ig) [38]. Analysis of the cultures, showed both a loss of p24 antigen and viral RNA. Thus, the treatment “purged” the cells of detectable viral antigen and RNA. Moreover, overnight treatment of HIV+ FDC with sCD21-Ig prior to addition of healthy, activated CD4 T cells blocked viral transmission and disrupted infection. We speculate that the decoy receptor acts by competing for the C3d-opsonized virus as it cycles to the cell surface of the FDC (see model in Fig 5). In this manner, C3d opsonized viral complexes bound via CD21 receptors are eliminated from the FDC as they cycle to the cell surface. These findings support a mechanism for viral retention in which CD21 binds and internalizes C3d-opsonized virus. They also suggest that sCD21-Ig could provide an adjunct therapy to ART or could be used in combination with bNab and provide a potential component of new therapeutic strategies to achieve a functional cure or viral eradication in ART-treated HIV-infected humans.

In summary, we hypothesize that C3-opsonized HIV particles are transported to the FDC by B cells, as has been shown with immune complexes, where it is taken-up and cycled in a neutral pH compartment until it encounters a CD4+ TFH (Fig 5). TFH have been described as the cell type most infected by HIV and are attracted to the FDC by a CXCL13 gradient [5,6]. Although the transfer event has not been studied here, we propose a mechanism in which the integrins LFA and VLA4 on the TFH can bind to ICAM and VCAM, respectively, on the FDC [47]. This would provide the proximity needed for the interaction of gp120 with CD4 and CCR5.

## Methods

### Mice

C57BL/B6 background mice were purchased from Jackson Laboratories and maintained in specific- pathogen-free facilities at Boston Children’s Hospital Program in Cellular and Molecular Medicine (PCMM), Harvard Medical School.

### Human subjects

Research conformed to ethical guidelines established by the ethics committee of the Massachusetts General Hospital, University of Montreal Health Center. Inguinal lymph nodes were excised under anesthesia by a surgeon at Massachusetts General Hospital according to normal surgical procedures. Tissue was then transported on ice to the BL2+ facility at the Ragon Institute. The committee on microbiological procedures at Harvard Medical School approved the study. Protocol number 03097.

### Ethics statement

Studies in mice were carried out in accordance with the recommendations in the Guide for the Care and Use of Laboratory Animals of the National Institutes of Health and approved by the Institutional Animal Care and Use Committees (IACUC) at Harvard Medical School and the Program in Cellular and Molecular Medicine (PCMM) at Boston Children’s Hospital. Approval numbers 889 and 04156.

Written, informed consent, approved by the Partners Human Research Committee of the Massachusetts General Hospital, was provided and signed by study participants before enrolment in the study. Protocol number: 2012P001806.

### Immune complex generation

B-Phycoerythrin (PE) (Anaspec) was used as a model Ag. ICs were generated by mixing 5 μg of PE, 5 μg of rabbit anti-PE IgG (Rockland), and 10% freshly isolated C57BL/B6 or human serum (as a source of complement) in 100 μl GVB++ buffer (Complement Tech) for 30 min at 37°C. Splenocytes from a C57BL/B6 mouse were then incubated with the immune complex mix for 30 min at 37°C to generate IC-bound B cells. Alternatively, the human B cell line Raji was used.

### FDC isolation and ex vivo culture

FDC isolation and culture procedures were modified from that described to be compatible with human [26]. In short, inguinal LNs were surgically excised from volunteer subjects or mice and digested at 37°C with 0.2 mg/ml collagenase, 1 mg/ml dispase and 10 μg/ml DNase I (Roche) for approximately 1 hour (digestions was started within one hour of excision, LNs were kept on ice in media). Then a single cell suspension was made by passing the suspension through a series of fire-polished pipets. CD45 cells were depleted and FDCs were enriched with magnetic bead sorting (Stem cell technologies) according to manufacturer’s protocol, with 50 μg anti-CD45 magnetic beads (Miltenyi) and with 50 μg biotinylated anti-CD35 antibody (Biolegend) followed by magnetic bead streptavidin (Miltenyi). Positively selected FDCs were resuspended in FDC media (RPMI supplemented with 10% FBS, 20 mM HEPES buffer, 0.2 mM MEM nonessential amino acids, 2 mM L-glutamine, and 1 mg/ml gentamicin) and plated on collagen-coated (5μg/cm^2^ Rat tail derived collagen; Roche) glass coverslips (#1.5, 25 mm diameter, Warner Instruments). 3–5 day cultured FDCs were used for different assays.

### ddPCR

HIV-1 and CD4 RNA was quantified using the QX100 Droplet Digital PCR system (Bio-Rad, Pleasanton, CA). The HIV-1 ddPCR mix consisted of: 10 μl 2x ddPCR super mix for probes (Bio-Rad); 500 nM of Forward (5’-CATGTTTTCAGCATTATCAGAAGGA-3’) and Reverse (5’-TGCTTGATGTCCCCCCACT-3’) primers; 250 nM probe mix (5’-FAM-CCACCCCACAAGATTTAAACACCATGCTAA-TAMRA-3’) and 3 μl of the cDNA into a final volume of 20 μl. The CD4 ddPCR mix consisted of: 10 μl 2x ddPCR super mix for probes (Bio-Rad); 500 nM of Forward (5’-AGTCCTCACACAGATACGCC-3’) and Reverse (5’-ACTCACATCCGAACACTAGCAA-3’) primers; 250 nM probe mix (5’-FAM-TGAAGTGGAGGACCAGAAGGAGGA-TAMRA-3’) and 3 μl of the cDNA into a final volume of 20 μl. The total mix was placed into the 8-channel cartridge, 70 μl of droplet generating oil was added and droplets were formed in the QX100 droplet generator (Bio-Rad). Droplet in oil suspensions were transferred to a 96 well plate and placed into the T100 Thermal Cycler (Bio-Rad). Cycling conditions were as follows: 95°C for 10 min, followed by 45 cycles of 94°C for 30 seconds and 60°C for 60 seconds. Subsequently, the droplets were automatically read by the QX100 droplet reader (Bio-Rad) and the data was analyzed with the QuantaSoft analysis software 1.3.2.0 (Bio-Rad).

### Immunohistochemistry

FDCs were fixed in 1% paraformaldehyde solution. Fixed samples were pre-incubated with anti- FcR (2.4G2, house produced) and 2% bovine serum albumin for blocking nonspecific and Fc-mediated binding. Anti-PE (Rockland), donkey anti-rabbit DyLight 405, anti-CD35, anti-Lamp-1 (Biolegend), fluorescent transferrin and anti-p24 (KC57-RD1, Beckman-Coulter) were used for staining ex vivo cultures of FDCs. Images were acquired with a FluoView FV1000 confocal microscope (Olympus) with a 20x lens (NA: 0.7) or a 60x water immersion lens (NA: 1.2) and processed with FluoView software (Olympus). Data were analyzed with Fiji software.

### Broadly neutralizing antibodies and soluble CD21-Ig

The broadly neutralizing antibodies used in this study are available through aidsreagent.org and were obtained through our collaboration with the Ragon Institute. VRC01, PG16 and PGT121 all have the human IgG1 isotype. sCD21-Ig consists of a full murine IgG1 antibody (hybridoma B1-8) bearing the two N-terminal CD21 SCR (short consensus repeat) domains that bind human C3d [38]. As isotype control the B1-8 IgG1 hybridoma was used.

## Supporting Information

S1 Fig Mouse FDCs retain HIV. (A) PBS (vehicle) or live GFP-HIV (green) was injected in the footpad of a mouse. After 48h the LN was collected, fixed and cryosectioned. Sections were stained with the FDC marker 8C12 (red). Note the dendritic pattern of FDC (red) within the follicles and the co-localization of HIV particles (green) with dendrites in HIV treated mice. Representative image. Each symbol represents a quantified follicle. (B) Quantification of the correlation of HIV-GFP signal with FDC signal was performed using the Cell Profiler software. Each symbol represents a different follicle; full z-stacks were analyzed per follicle. ***P<0.001 (unpaired Student’s *t* test) n = 3.
(TIF)

S2 Fig Line profile analysis of HIV+ vesicles. Line profiles are fluorescent intensity measurements over a line in an image, creating a graph. (A) Lines over the HIV vesicle in XY and YZ used to calculate the line profiles of all 3 stains. (B) Line profile graphs from the lines shown in A in XY and YZ. Results confirm co-localization of transferrin signal with p24 staining in both XY and YZ optical planes.
(TIF)

S3 Fig PEIC can be displaced from human FDCs using sCD21-Ig. (A) Human FDCs were loaded with PEIC using Raji B cells in vitro, then FDCs were either mock treated with isotype control IgG (left panel) or sCD21-Ig treated (right panel) overnight and analyzed the following day. After sCD21-Ig treatment no PEIC+ vesicles were detected. By contrast, PEIC+ vesicles were identified in the isotype treated controls. (B) The mean fluorescent intensity (MFI) of 16 random fields of views (FOV) at lower magnification was compared. ****p<0.0001 (unpaired Student’s *t* test).
(TIF)

S4 Fig Schematic of experimental procedure. LNs were harvested and FDCs isolated, first by depletion of CD45+ B and T cells, followed by positive selection for CD35+ cells. Cultures were either treated with sCD21-Ig, isotype control or RNA was directly isolated. The remaining samples were washed and HIV negative CD4 T cells were added to the FDC wells and co-cultured for 5 days. Afterwards FDCs and T cells were separated and RNA isolated. HIV RNA was quantified by ddPCR. Numbers in the graph represent total virions per well. The average cell number per well for this subject was between 15k and 150k (subject #10).
(TIF)

S5 Fig LN cells from one subject were analyzed by flow cytometry to determine the amount of CD35 positive T cells and to assess the contamination of CD4 positive T cells during CD35 magnetic bead positive selection. (A) CD35 expression on T and B cells shows expression on >96% of B cells as expected, interestingly 1% of T cells also express CD35, although the mean fluorescent intensity levels were lower than on B cells. (B) Before the positive CD35 bead selection for FDCs >79% of cells were CD3 positive. In contrast, after CD35 positive selection for FDC, only 0.3% (estimate 1 cell) was CD3 positive. Thus, the contribution of T cells to the CD35 positively selected FDC cultures, is negligible based on immune staining and flow cytometry.
(TIF)

S6 Fig In order to determine if magnetic bead sorted-FDC in our historical samples were contaminated with CD4 T cells, we quantified CD4 mRNA levels in our FDC samples. To correlate mRNA levels with cellular contamination, we prepared a standard curve by sorting B and T cells separately and then spiking the B cells with known numbers of T cells (0 to 10^6^ T cells in 10-fold increments, 7 data points). RNA was extracted from the B cell samples bearing a known number of contaminating CD4 T cells and the amount of CD4 mRNA quantitated using ddPCR and a standard curve prepared. (A) Standard curve of CD4 mRNA versus CD4 T cell number. (B) Extrapolation of the number of CD4 T cells in samples based on CD4 mRNA levels. Extrapolation of the number of CD4 T cells in a pure CD4 sample correlates with the number of T cells analyzed (approximately 10^5^ cells). CD4 mRNA levels in the FDC samples indicate limited CD4 T cell contamination of the historical FDC samples. ***p<0.001 (unpaired Student’s *t* test). n = 6 subjects. (C) Lymphocytes (10^5^) from the LN of one HIV^+^ subject were FACS sorted and RNA was isolated as described. Then ddPCR was performed in order to determine the HIV RNA copy number. These data show that very few lymphocytes in the LN retain HIV RNA.
(TIF)
