# Supplementary figures and images for: Follicular Dendritic Cells Retain Infectious HIV in Cycling Endosomes
Source: PLoS Pathog. 2015 Dec 1;11(12):e1005285. doi: 10.1371/journal.ppat.1005285 (PMC4666623; doi:10.1371/journal.ppat.1005285)

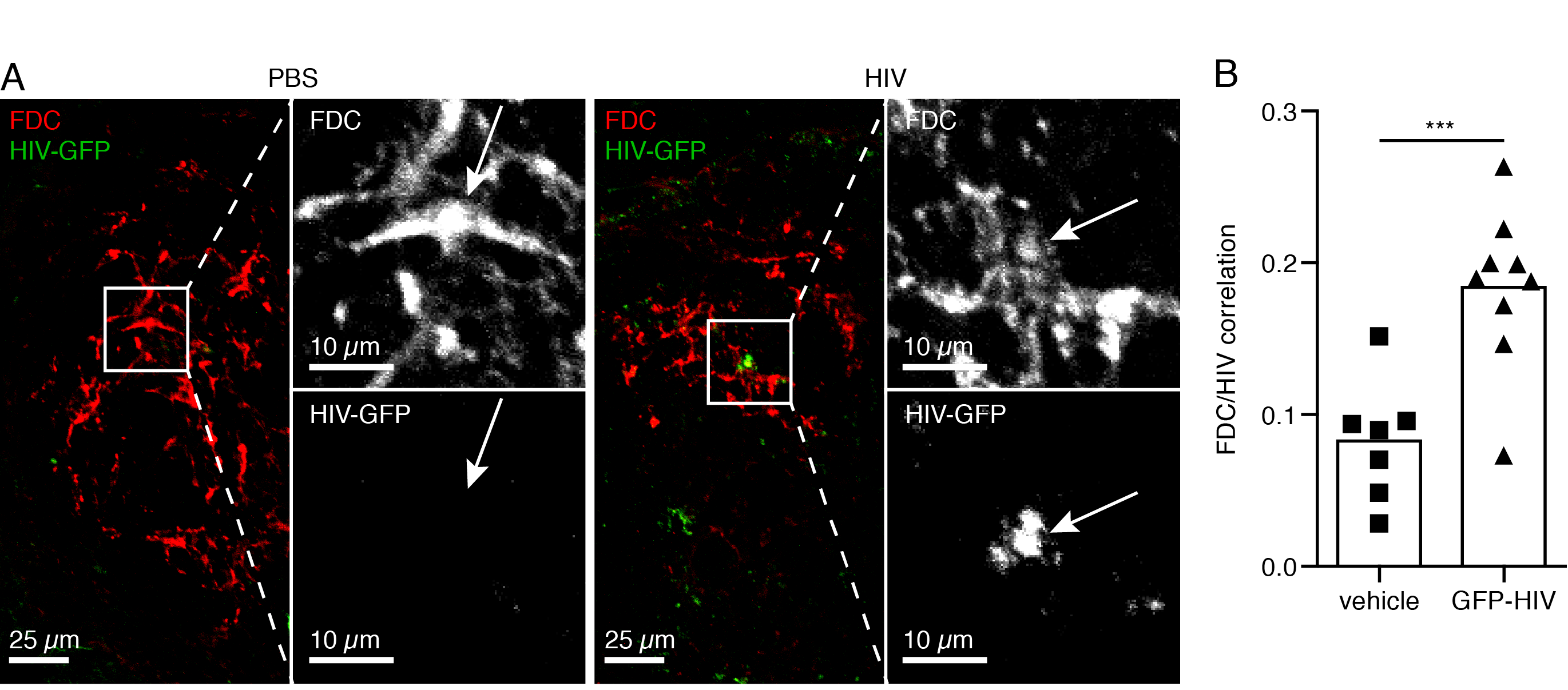

Supplement: S1 Fig — (A) PBS (vehicle) or live GFP-HIV (green) was injected in the footpad of a mouse. After 48h the LN was collected, fixed and cryosectioned. Sections were stained with the FDC marker 8C12 (red). Note the dendritic pattern of FDC (red) within the follicles and the co-localization of HIV particles (green) with dendrites in HIV treated mice. Representative image. Each symbol represents a quantified follicle. (B) Quantification of the correlation of HIV-GFP signal with FDC signal was performed using the Cell Profiler software. Each symbol represents a different follicle; full z-stacks were analyzed per follicle. ***P<0.001 (unpaired Student’s t test) n = 3. (TIF) [file ppat.1005285.s001.tif]

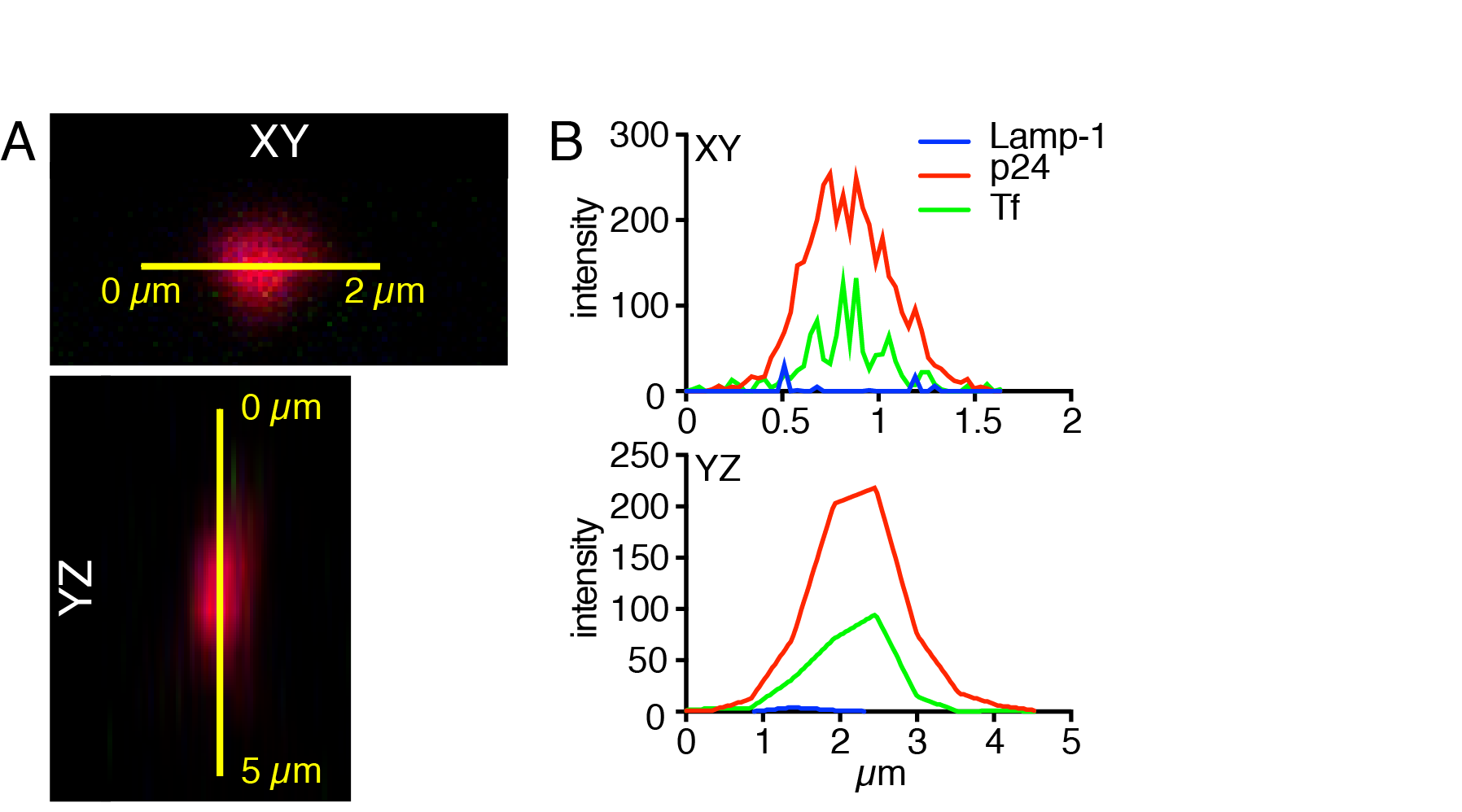

Supplement: S2 Fig — Line profiles are fluorescent intensity measurements over a line in an image, creating a graph. (A) Lines over the HIV vesicle in XY and YZ used to calculate the line profiles of all 3 stains. (B) Line profile graphs from the lines shown in A in XY and YZ. Results confirm co-localization of transferrin signal with p24 staining in both XY and YZ optical planes. (TIF) [file ppat.1005285.s002.tif]

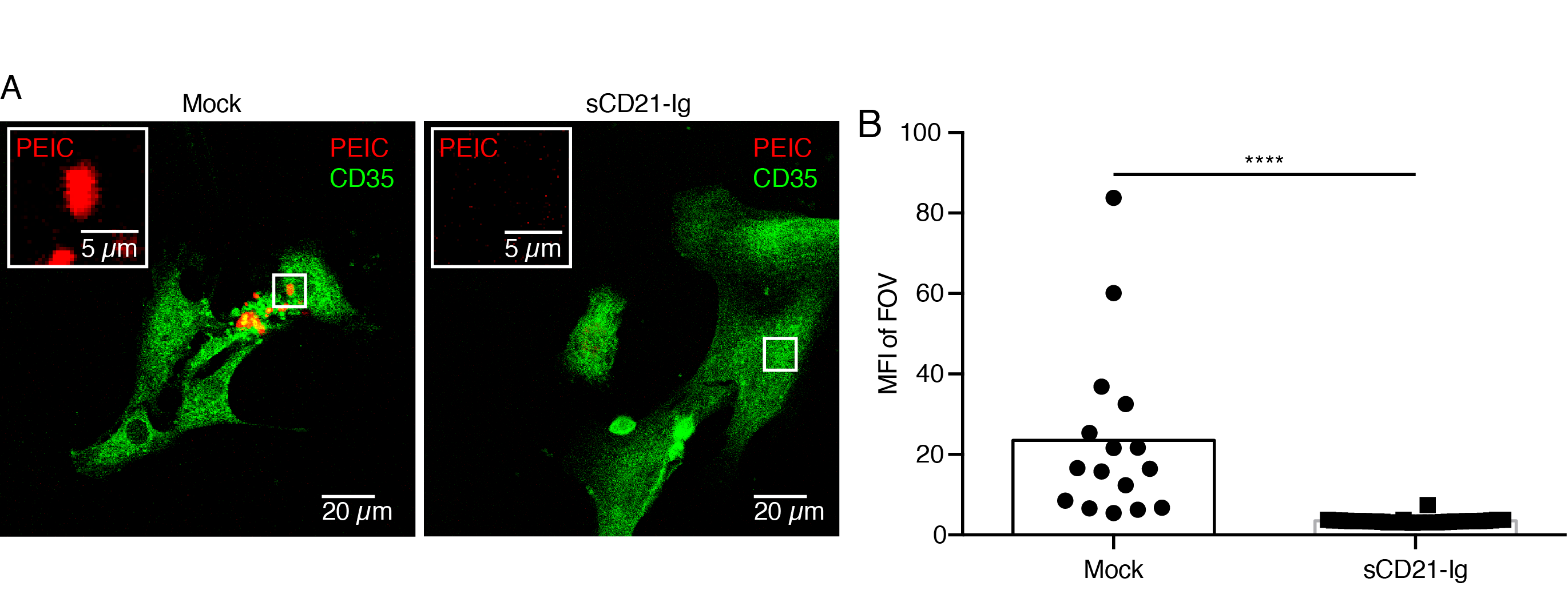

Supplement: S3 Fig — (A) Human FDCs were loaded with PEIC using Raji B cells in vitro, then FDCs were either mock treated with isotype control IgG (left panel) or sCD21-Ig treated (right panel) overnight and analyzed the following day. After sCD21-Ig treatment no PEIC+ vesicles were detected. By contrast, PEIC+ vesicles were identified in the isotype treated controls. (B) The mean fluorescent intensity (MFI) of 16 random fields of views (FOV) at lower magnification was compared. ****p<0.0001 (unpaired Student’s t test). (TIF) [file ppat.1005285.s003.tif]

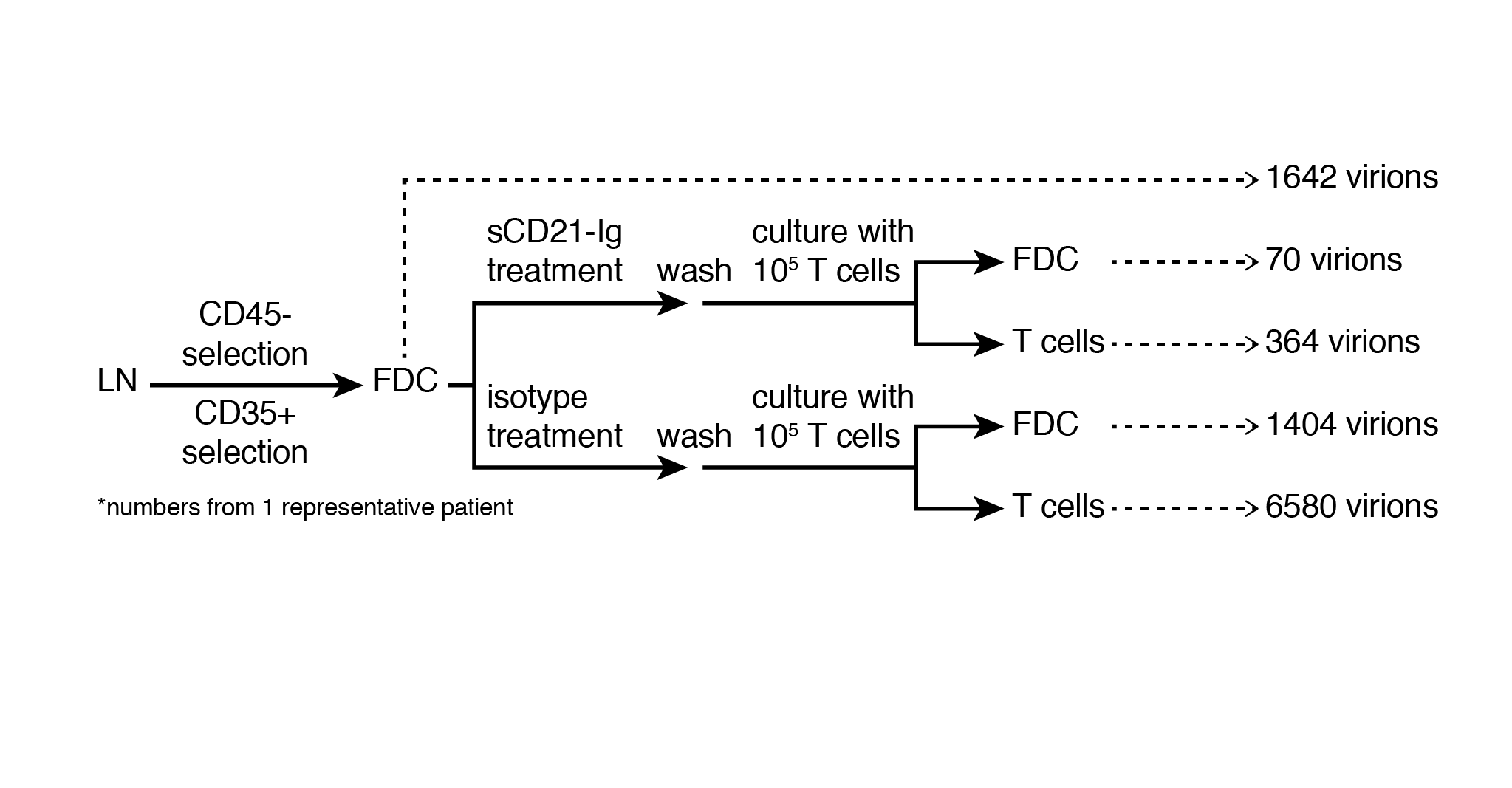

Supplement: S4 Fig — LNs were harvested and FDCs isolated, first by depletion of CD45+ B and T cells, followed by positive selection for CD35+ cells. Cultures were either treated with sCD21-Ig, isotype control or RNA was directly isolated. The remaining samples were washed and HIV negative CD4 T cells were added to the FDC wells and co-cultured for 5 days. Afterwards FDCs and T cells were separated and RNA isolated. HIV RNA was quantified by ddPCR. Numbers in the graph represent total virions per well. The average cell number per well for this subject was between 15k and 150k (subject #10). (TIF) [file ppat.1005285.s004.tif]

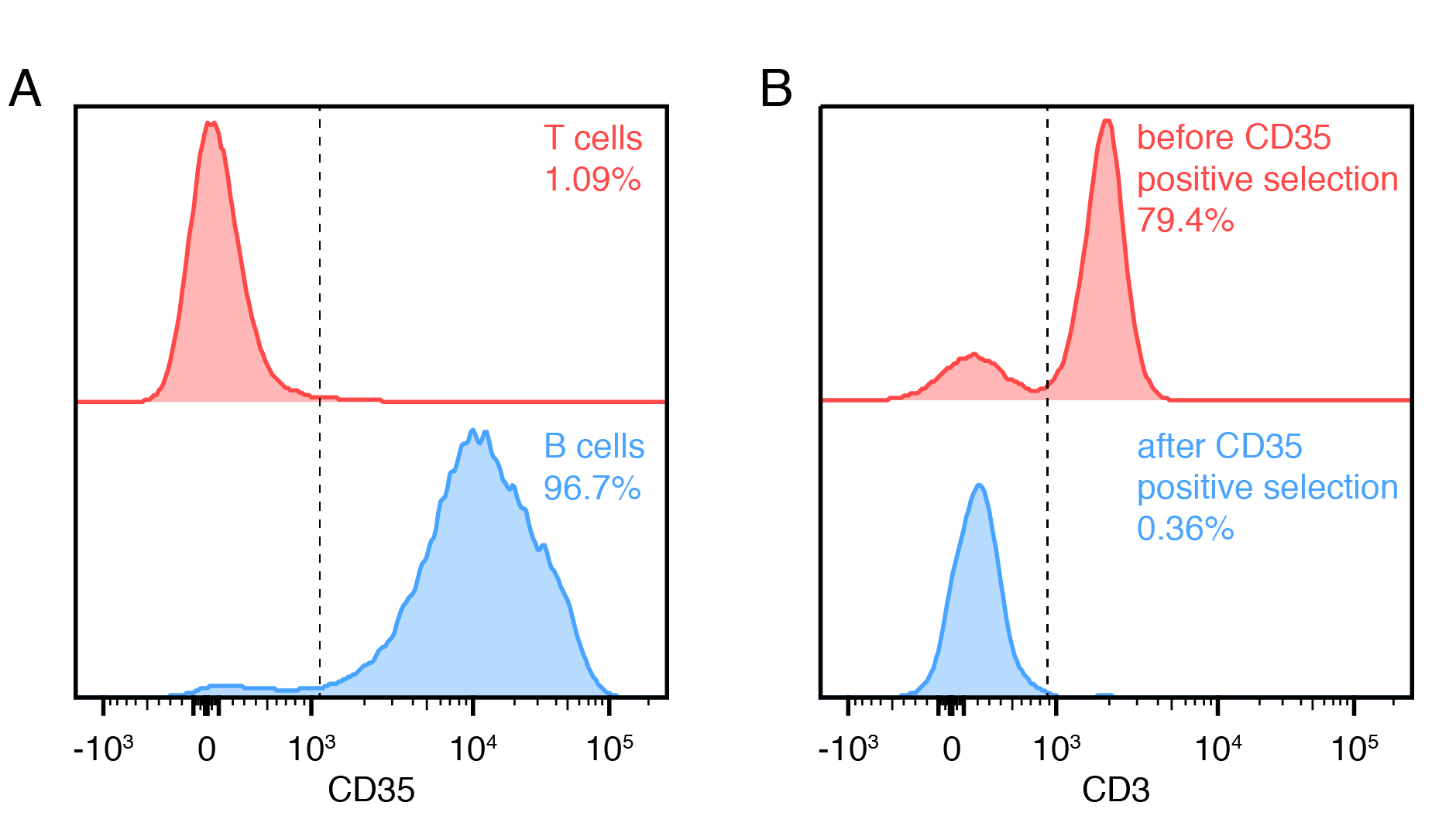

Supplement: S5 Fig — (A) CD35 expression on T and B cells shows expression on >96% of B cells as expected, interestingly 1% of T cells also express CD35, although the mean fluorescent intensity levels were lower than on B cells. (B) Before the positive CD35 bead selection for FDCs >79% of cells were CD3 positive. In contrast, after CD35 positive selection for FDC, only 0.3% (estimate 1 cell) was CD3 positive. Thus, the contribution of T cells to the CD35 positively selected FDC cultures, is negligible based on immune staining and flow cytometry. (TIF) [file ppat.1005285.s005.tif]

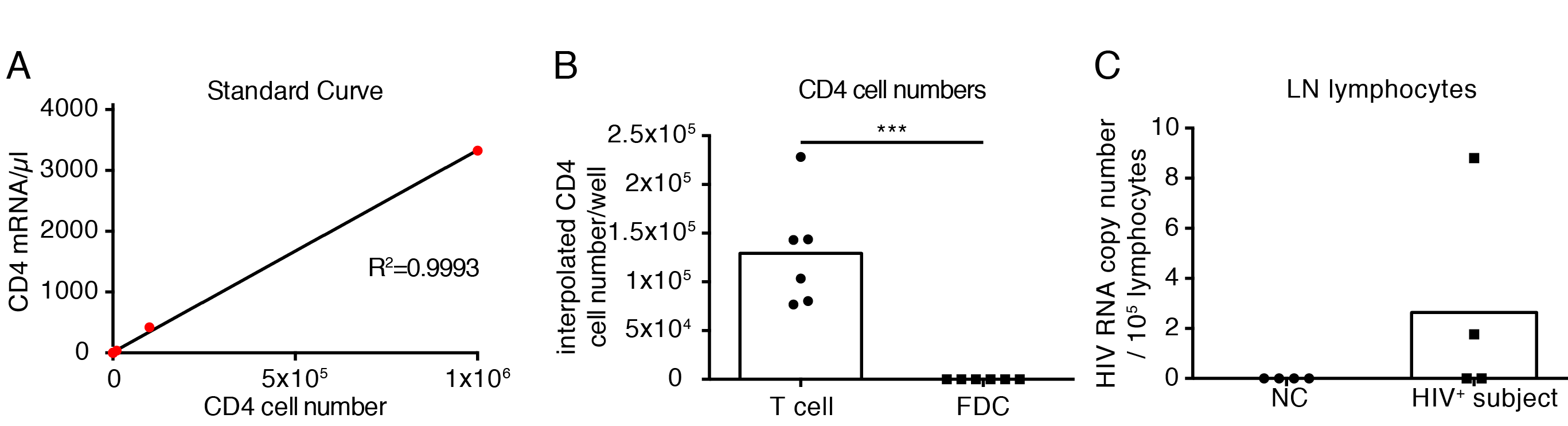

Supplement: S6 Fig — To correlate mRNA levels with cellular contamination, we prepared a standard curve by sorting B and T cells separately and then spiking the B cells with known numbers of T cells (0 to 106 T cells in 10-fold increments, 7 data points). RNA was extracted from the B cell samples bearing a known number of contaminating CD4 T cells and the amount of CD4 mRNA quantitated using ddPCR and a standard curve prepared. (A) Standard curve of CD4 mRNA versus CD4 T cell number. (B) Extrapolation of the number of CD4 T cells in samples based on CD4 mRNA levels. Extrapolation of the number of CD4 T cells in a pure CD4 sample correlates with the number of T cells analyzed (approximately 105 cells). CD4 mRNA levels in the FDC samples indicate limited CD4 T cell contamination of the historical FDC samples. ***p<0.001 (unpaired Student’s t test). n = 6 subjects. (C) Lymphocytes (105) from the LN of one HIV+ subject were FACS sorted and RNA was isolated as described. Then ddPCR was performed in order to determine the HIV RNA copy number. These data show that very few lymphocytes in the LN retain HIV RNA. (TIF) [file ppat.1005285.s006.tif]
